# Prognostic Significance of Lymph Node Ratio in Ovarian Cancer

**DOI:** 10.1515/med-2019-0024

**Published:** 2019-03-02

**Authors:** Xiaoxia Tong, Haoran Li, Huiqing Chen, Dong Zhai, Yangyang Pang, Ruyin Lin, Yuan Xu

**Affiliations:** 1 Second Affiliated Hospital, Fujian Medicine University, 34 Zhongshan Road, Licheng,Quanzhou, 362000, China; 2 Cancer Institute, Fudan University Shanghai Cancer Center, Department of Oncology, Shanghai Medical College, Fudan University Shanghai 200032, China; 3 Department of gynaecology and obstetrics, Second Affiliated Hospital, Fujian Medicine University, 34 Zhongshan Road Licheng,Quanzhou, 362000, China; 4 Third Affiliated Hospital of Zhejiang Chinese Medical University, Hangzhou 310005, China; 5 Jiading Central Hospital, Shanghai 201800, China

**Keywords:** Lymph node, Serous epithelial ovarian cancer, Overall survival

## Abstract

Lymphadenectomy is critical in the clinical prognosis of ovarian cancer patients. Therefore, we assessed whether lymph node ratio (LNR) has predictive value on overall survival (OS) of patients with serous epithelial ovarian cancer (SEOC). A total of 7,815 eligible SEOC patients were identified from the Surveillance, Epidemiology, and End Results (SEER) database, who underwent surgical resection between 1973 and 2013. We used the time-dependent receiver operating characteristic (ROC) curve and the area under curve to determine the optimal cut-off value of LNR. The predictive role of LNR was analyzed by Cox proportional hazards regression model. The effects of LNR and positive lymph nodes (PLN) on OS were evaluated by comparing the time-dependent ROC curves. The time-dependent ROC curves showed that the optimal LNR cut-off value was 42.0% for nodal-positive SEOC. As shown in Kaplan-Meier survival curves, survival was significantly poorer for all patients with LNR≥42.0% (log-rank test: P<0.0001), regardless of the stage. In the multivariate Cox analysis, LNR≥42.0% remained a significant and independent predictor of mortality risk for all patients [hazards ratio: 1.526, 95% confidence interval: 1.415-1.647; P<0.0001], compared with those LNR<42.0%. These results suggest that LNR, rather than the number of PLN or stage, could be regarded as a promising predictor of mortality risk, particularly in stage-III SEOC patients.

## 1 Introduction

According to GLOBOCAN 2012, the incidence of global ovarian cancer was 3.6% and the mortality rate reached up to 4.3% [5]. In the United States, with estimated 22,440 new diagnosed cases and 14,080 deaths of ovarian cancer patients in 2017[1], epithelial ovarian cancer (EOC) became the leading cause of deaths among gynecological malignancies, ranking fifth in mortality rates among all types of cancers [1,2]. Previous studies have demonstrated that different histological subtypes of ovarian cancer lead to distinct clinical characteristics and prognosis [5]. Serous epithelial ovarian cancer (SEOC), which accounts for 80-90% ovarian cancer patients, is the most common type of all EOC patients [11]. Due to the dismal 5-year survival rate, it is important to establish prognostic factors for SEOC to better evaluate survival for proper clinical management.

Currently, the standard treatment of ovarian cancer patients was primary debulking surgery, followed by platinum and taxane-based adjuvant chemotherapy. The volume of residual disease was the most important independent prognostic factor of prognosis [4,20]. Several studies have investigated the role of CA-125, imaging and laparoscopy, as well as genetic signature in predicting the outcome of surgery. However, there were still no ideal index to predict outcomes of surgery due to its irreproducible.

According to the International Federation of Gynecology and Obstetrics (FIGO), complete pelvic and para-aortic lymphadenectomy was recommended for primary debulking surgery. However, radical lymph node (LN) dissection was associated with serious morbidity [3]. In addition, LN status is regarded as an important factor for ovarian cancer staging in FIGO staging system. Previous studies have revealed that the LN status significantly affects the prognosis of patients with ovarian cancer [10 ,8]. Although the lymphadenectomy could improve the survival of patients with advanced EOC [1], the information of positive lymph nodes (PLN) resected provided by lymphadenectomy could not fully illustrate the real status of LNs. Accordingly, the ratio of metastatic lymph nodes (LNR) has been proposed, calculating as the ratio of the number of PLN to the total number of LNs resected. Therefore, LNR has been acknowledged as an independent predictor of mortality risk for cancers of the esophagus [1,3], stomach [6,11], colorectum [6] and ovaries. However, the patients included in previous EOC studies were mostly staged as FIGO IIIC or/and IV, containing different histological types.

In the present study, under the hypothesis that LN metastasis varies by different histological types of EOC, we focused the prognostic value of LNR on overall survival (OS) among SEOC patients using a population-based Surveillance, Epidemiology and End Results (SEER) database.

## 2 Methods

### 2.1 Data sources

Patients with a pathological diagnosis of primary SEOC were identified from the 18 population-based registries in the SEER database. The National Cancer Institute’s SEER*Stat software (version 8.1.2; Surveillance Research Program, www.seer.cancer.gov/seerstat) was used to identify eligible SEOC patients diagnosed between 1973 and 2013.

All the patients with positive LNs after lymphadenectomy were included. The histology code of ICD-O-3 for SEOC has four major histological subtypes (i.e., serous, mucinous, clear cell and endometrioid). From the SEER database, the detailed clinicopathologic information about age at diagnosis, race, differentiated grade, lateral status, radiation treatment, AJCC staging (AJCC 7th), number of examined lymph nodes, number of pathologically PLNs and survival information were extracted for further analysis. Finally, the patients were re-staged by following the rules of the FIGO staging system.

### 2.2 Statistical analysis

The univariate Cox proportional hazards regression model was used to identify predictors of OS. The optimal cut-off value of LNR was determined by the time-dependent receiver operating characteristic (ROC) curve. With the optimal LNR cut-off value, Kaplan-Meier curves were constructed, and the log-rank test was used to estimate the univariate significance of the LNR for the stage-III and -IV patients, respectively. To evaluate the impact of LNR on survival, Cox regression analysis was performed to determine the hazards ratios (HRs) and their 95% confidence interval (95% CI). Moreover, the effects of LNR and PLN on OS were compared by the time-dependent ROC curves and area under the curve (AUC). All statistical tests were two-sided, and a *P*value <0.05 was considered statistically significant.

The statistical analysis was performed by SAS (version 9.4, University of North Carolina, USA) and R (version 3.2.3, R Foundation, Vienna, Austria) software, unless otherwise stated.

All procedures were performed in accordance with the Helsinki Declaration (1964) and its later amendments or comparable ethical standards.

## 3 Results

### 3.1 Patients’ characteristics

A total of 7,815 eligible patients with SEOC were included for the final analysis. Basic characteristics of the patients are shown in Table 1. According to the FIGO staging system, of all the patients, 43.5% were of stage-III and 19.3% were of stage-IV, but the remaining 2,904 patients (37.2%) were not given a specific FIGO staging, but were in fact either in stage-III or stage-IV based on the their actual nodal status. By the extent of LNs dissected, patients were then divided into three groups to examine their associations with survival (1-10 LN, 11-20 LN and >20 LN groups). The results showed that the mortality risk decreased as the number of LNs dissected increased (P<0.0001), which may be regarded as a protective factor of prognosis. As expected, we found a decreasing mortality risk for an increasing number of PLN examined among the three groups (1 LN, 2-4 LN and >4 LN groups) in a dose-response manner.

**Table 1:** Baseline characteristics and associations with overall survival in SEOC patients

Variables	No. (%)	Univariate Cox analysis HR (95% CI)	P value
Age (years)			
<60	3,790 (48.5)	1.00	
≥60	4,025 (51.5)	1.596 (1.508-1.690)	<0.0001
Race			
White	6,830 (87.4)	1.00	
Black	451 (5.8)	1.058 (0.937-1.195)	0.363
Asian / Pacific Islander	473 (6.1)	0.804 (0.708-0.914)	0.001
Others	61 (0.8)	0.976 (0.689-1.382)	0.891
FIGO staging			
III	3,401 (43.5)	1.00	
IV	1,510 (19.3)	1.655 (1.524-1.797)	<0.0001
III/IV*	2,904 (37.2)	1.343 (1.258-1.435)	<0.0001
Grade			
Well differentiated	262 (3.3)	1.00	
Moderately differentiated	939 (12.0)	2.460 (2.002-3.024)	<0.0001
Poorly differentiated	3,974 (50.9)	2.672 (2.201-3.244)	<0.0001
Undifferentiated/anaplastic	1,622 (20.8)	2.778 (2.260-3.414)	<0.0001
Unknown	1,018 (13.0)	2.499 (2.032-3.073)	<0.0001
Lateral status			
One site	2,640 (33.8)	1.00	
Paired site	261 (3.3)	1.691 (1.300-2.201)	<0.0001
Bilateral site	4,914 (62.9)	1.131 (1.065-1.202)	0.611
Radiation treatment			
No	7,637 (97.7)	1.00	
Yes	132 (1.7)	0.988 (0.807-1.211)	0.911
Unknown	46 (0.6)	0.908 (0.626-1.316)	0.609
Extent of LNs dissected (No.)			
1-10	4,256 (54.5)	1.00	
11-20	1,774 (22.7)	0.816 (0.760-0.876)	<0.0001
>20	1,785 (22.8)	0.745 (0.692-0.801)	<0.0001
Positive LNs examined (No.)			
1	2,772 (35.5)	1.00	
2-4	2,619 (33.5)	1.084 (1.013-1.161)	0.020
>4	2,424 (31.0)	1.195 (1.115-1.281)	<0.0001

Abbreviations: SEOC= Serous Epithelial Ovarian Cancer; HR=Hazards Ratio; FIGO= the International Federation of Gynecology and Obstetrics; LN=Lymph Node. *These patients were not given a specific FIGO staging in SEER database, they were either in stage III or stage IV based on their actual nodal status.

### 3.2 The optimal cut-off points of LNR

Using the time-dependent ROC curve, we estimated the optimal cut-off value of LNR in nodal-positive patients. The results indicated that 42.0% was the optimal LNR cut-off value for predicting OS. The AUC was 0.631 (*P*=0.0001) for stage-III patients (n=3,401), 0.514 (*P*=0.106) for stage-IV patients (n=1,510) and 0.607 for stages III/IV patients (n=2,904).

### 3.3 The prognostic impact of LNR on survival

**Figure 1:**
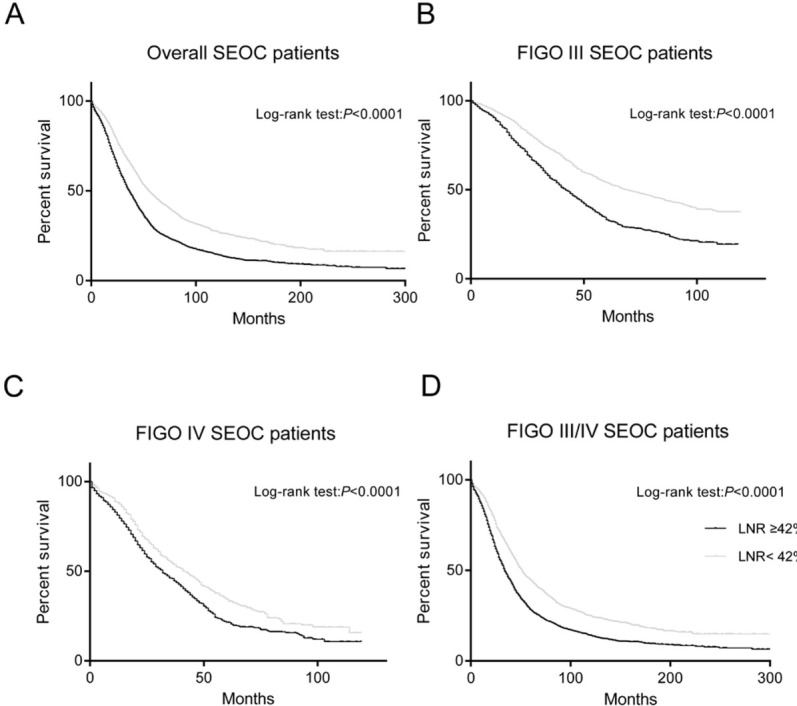
Kaplan-Meier curve for patients with LNR<42.0% and LNR≥42.0%. nodal-positive patients (A), stage-III patients (B), stage-IV patients (C) and stage III/IV patients (D), respectively. OS of patients with LNR≥42.0% was significantly shorter than that of those with LNR<42.0% (P < 0.0001, log-rank test).

**Figure 2:**
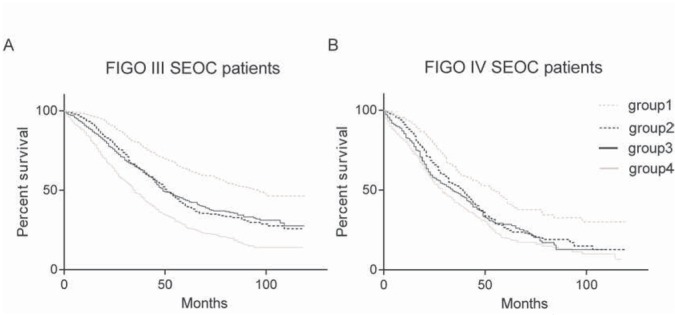
The Kaplan-Meier curve of LNR on overall survival by age (groups of age<60 and age≥60) for stage-III patients(A). The Kaplan-Meier curve of LNR on overall survival by age (groups of age<60 and age≥60) for stage-IV patients (B). (group1: age<60 and LNR<42%; group2: age<60 and LNR≥42%; group3: age≥60 and LNR<42%; group4: age≥60 and LNR≥42%)

Then, we divided patients into two groups by the optimal LNR cut-off value (groups of LNR<42.0% and LNR≥42.0%). Kaplan-Meier curves were constructed to reveal the effect of LNR on OS, and the results suggested that patients with LNR≥42.0% had a significantly poorer survival for all patients (log rank test: P<0.0001) (Figure 1B and 1C.) The Kaplan-Meier curve of LNR on OS by age (groups of age<60 and age≥60) was shown in Figure 2(A) and Figure 2(B)

In a multivariate Cox model, LNR≥42.0% was found to be a significant and independent predictor of unfavorable survival for all the patients after adjustment for age, race, grade, lateral status and radiation treatment (HR=1.526, 95% CI= 1.415-1.647 and P<0.0001) (Table 2). In the stratified analysis by stage, the HR was 1.682 (95% CI=1.508-1.875 and P=0.0001) for stage-III patients (n=3,401), 1.419 (95% CI=1.201-1.676 and P<0.0001) for stage-IV patients (n=1,510), and 1.540 (95% CI=1.384-1.712 and *P*<0.0001) for stages III/IV patients (n=2,904).

**Table 2:** Univariate and multivariate Cox proportion hazards regression analysis.

Stage	No. (%)	Univariate analysis HR (95% CI)	P value	Multivariate analysis* HR (95% CI)	P value
All patients (n=7,815)					
LNR<42.0%	3,608 (46.2)	1.00		1.00	
LNR≥42.0%	4,207 (53.8)	1.600 (1.510-1.694)	<0.0001	1.526 (1.415-1.647)	<0.0001
FIGO III (n=3,401)					
LNR<42.0%	1,785 (52.5)	1.00		1.00	
LNR≥42.0%	1,616 (47.5)	1.751 (1.580-1.940)	<0.0001	1.682 (1.508-1.875)	<0.0001
FIGO IV (n=1,510)					
LNR<42.0%	595 (39.4)	1.00		1.00	
LNR≥42.0%	915 (60.6)	1.368 (1.195-1.566)	<0.0001	1.419 (1.201-1.676)	<0.0001
FIGO III/IV (n=2,904)					
LNR<42.0%	1,228 (42.3)	1.00		1.00	
LNR≥42.0%	1,676 (57.7)	1.498 (1.382-1.625)	<0.0001	1.540 (1.384-1.712)	<0.0001

*Adjusted for age, race, grade, lateral status and radiation treatment. Abbreviations: HR= Hazards Ratio; LNR=Lymph Node Ratio

### 3.4 Comparisons the predictive value of LNR and PLN

**Figure 3:**
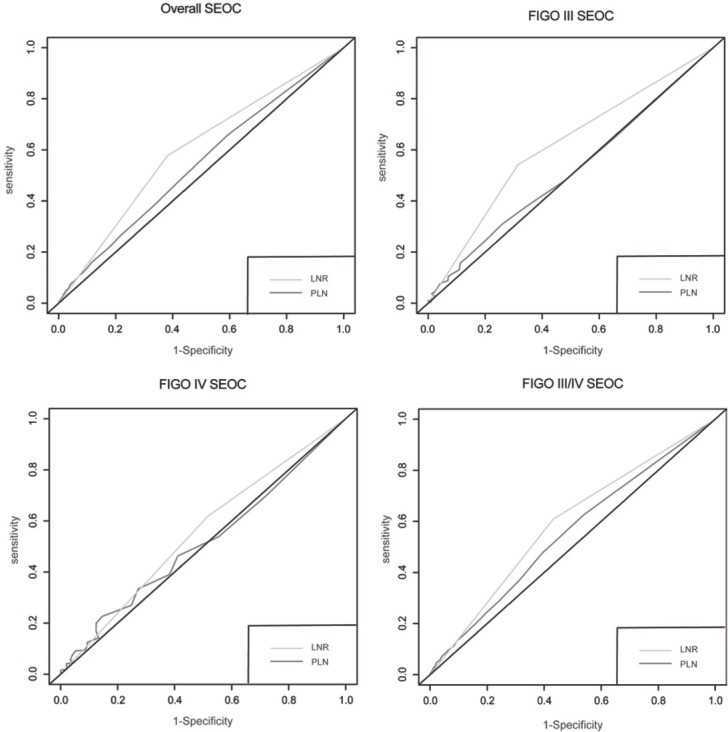
Survival prediction model under the comparison of LNR and PLN in SEOC patients of nodal positive (A), stage-III(B), stage-IV (C) and in stage-III/IV(D), respectively. The AUC for the LNR was higher than the number of PLN for predicting overall survival in SEOC patients

Furthermore, using the time-dependent ROC curves, LNR was revealed to be superior to the number of PLN in distinguishing prognosis for all nodal-positive patients (AUC 0.615 vs. 0.542, P<0.0001), stage-III patients (AUC 0.631 vs. 0.514, P<0.0001), and stage-III/IV patients (AUC 0.607 vs. 0.550, P=0.036) (Figure 3). For stage-IV patients, LNR still had a better performance than the number of PLN in discriminating OS, although the results did not reach a statistical significance (AUC 0.514 vs.0.511, P=0.565).

## 4 Discussion

Although previous studies have identified the function of LNR on EOC, our study focused on the prognostic value of LNR in advanced LN-positive SEOC patients. Our results as anticipated, demonstrated that LNR could be regarded as a promising predictor of mortality risk for LN positive patients, and its prognostic value was superior to that of PLN. In addition, we confirmed that 42.0% of LNR was an optimal predictor of clinical prognosis for SEOC patients.

LNR, as a prognostic factor, has been illustrated in multiple types of cancers such as; cancers of the breasts, stomach, pancreas, colon and ovaries. To date, several studies have revealed the prognostic value of LNR for ovarian cancer survival. Mahdi et al. reported that a greater number of LNR was independently associated with a poorer survival in 6,310 patients with stage-III/IV EOC [1] . Moreover, Zhou et al. demonstrated the prognostic value of LNR on survival for patients with stage-IIIC EOC from the SEER database [11]. Furthermore, the association of LNR with survival was also reported by some studies with smaller samples sizes [3,8]. Consistent with previous studies, we confirmed LNR as a reliable prognostic value for mortality risk in SEOC patients with positive nodes dissected as we used in the re-staging the patients.

In contrast to previous studies, the present study uniquely focused on SEOC patients, which has the potential to reduce bias introduced by variation of different histological types. It has been proved that patients with different histological types have different probabilities of LNs metastasis [2]; for instance, patients with serous and clear cell tumors were more likely to have LN involvement when compared with other histological types [1,2]. Serous tumors have two to three fold risk of developing LN metastasis in comparison with non-serous tumors [2]. Given the diverse status of LN involved, it would be valuable to determine the effect of LNR on mortality risk in SEOC patients. Our results showed that LNR had a better capability than the number of PLN in predicting OS for patients with stage-III SEOC; therefore, it could be used as a better optimal marker to guide the postoperative decision and prognostic evaluation and management. Furthermore, the results indicated that 42.0% of LNR, the optimal cut-off value by time-dependent ROC curves, could better discriminate mortality risk of patients with stage-III SEOC. However, the present study did not find the preferential value of LNR in stage-IV patients, which may result from the limited sample size and should be confirmed by further studies with larger sample sizes. Another reason is that stage-IV patients are at the highest mortality risk, no matter how many LNs have been dissected; therefore, the predictive ability of PLN and LNR might disappear as a whole or in part due to the rapid progression of disease.

There are several limitations in the present study. The main limitation is the inherent bias that exists in retrospective studies. In addition, we lack information on adjuvant chemotherapy and the exact locations of LNs dissected. However, the strength of the present study was that we focused on single histological type --serous EOC, which has a distinguished clinical performance and prognosis. With the optimal cut-off value of LNR, we revealed that a higher LNR could predict a poor survival in patients with stage-III/IV SEOC, which may be a useful clinical predictor. Although the AUC of the cut-off value of LNR were not persuasive enough to discriminate OS (ranged from 0.607 to 0.631), the value of LNR could be more significant in predicting prognosis as an ancillary index for patients that underwent lymphadenectomy, and the cut-off value could assist surgeons in making clinical decision and performing postoperative evaluation and management.

In conclusion, our results showed that a higher LNR was associated with poor survival in SEOC patients from the population-based SEER database. In particular, the optimal LNR cut-off value of 42.0% is a significant and independent predictor of mortality risk, most likely in patients with stage-III SEOC, which is superior to the number of PLN.
